# Age and Proactive Interference in Visual Working Memory: Reassessing the Recent-Probes Task

**DOI:** 10.3390/brainsci16080848

**Published:** 2026-08-10

**Authors:** Tom Mercer

**Affiliations:** Centre for Psychological Research, University of Wolverhampton, Wolverhampton WV1 1LY, UK; t.mercer2@wlv.ac.uk; Tel.: +44-(0)1902-321368

**Keywords:** aging, visual working memory, proactive interference, recent-probes task

## Abstract

**Highlights:**

**What are the main findings?**

- Older adults were not more sensitive to proactive interference in visual working memory than younger adults.
- Both younger and older adults were more prone to proactive interference when stimuli were unique and the response on the memory test was immediate.

**What are the implications of the main findings?**

- These data suggest that problems with visual working memory in older age may not be due to proactive interference.
- The findings challenge the view that older adults experience inhibitory deficits that makes proactive interference in visual working memory difficult to manage.

**Abstract:**

Background/Objectives: Proactive interference (PI) occurs when existing memories make it harder to retrieve newer information, and visual working memory in older adults may be impacted by increased vulnerability to PI. However, evidence supporting age-sensitive PI in the recent-probes task—a major technique for measuring PI—is mixed. Additionally, features of this procedure may have exaggerated or distorted the effect, so the current study reassessed the recent-probes task. Methods: Throughout trials in an online experiment, adults aged 18–29 and 64–80 memorized four images over a brief delay and then had to choose between a current target and a foil depicting an item from the previous trial (a recent negative probe) or a more distant trial (a non-recent negative probe). Participants were cued to respond either immediately or after a pause, and targets came from a small, extensively repeated set or a unique set. Results: PI was present, with slower and less accurate responding to RN than NRN foils, and errors notably increased when stimuli were unique and an immediate response was required. However, there was little evidence for age differences in PI. Conclusions: The present study did not suggest that older adults are more vulnerable to PI than younger adults, at least in this task and with this sample.

## 1. Introduction

Visual working memory (VWM) is responsible for maintaining and manipulating visuospatial information over brief periods of time. It plays a critical role in daily tasks, yet it does appear to decline with age, as shown in Brockmole and Logie’s [1] major study of over 55,000 participants. This study revealed a linear decline in VWM performance from the ages of 20 to 75, with the worst VWM in those aged 56 and older.

One factor that may impair VWM in older age is proactive interference (PI), which occurs when existing memories interfere with processing and retrieving current information [2]. PI may be more difficult to manage in older age, contributing to VWM difficulties, and this possibility has been investigated using the long-established recent-probes task, e.g., [3,4]. This procedure, an adapted version of the Sternberg recognition task [5], requires participants to remember an array of target objects over a brief delay. They must then determine whether a single probe matches any of the current targets. PI is manifested on mismatching trials, as probes that match targets from the *previous* trial (a Recent Negative/RN probe) typically increase errors and slow responding compared to probes that have not appeared recently (a Non-Recent Negative/NRN probe) [6]. This provides a behavioral measure of PI.

Some studies introducing visual stimuli into this task have found that older adults are particularly vulnerable to PI in VWM. Loosli et al. [3], using images of animals as the target set, reported that adults aged 66–74 made more errors on RN than NRN trials in comparison to adults aged 19–31. The same trend was observed for response time (RT), though this was only marginally significant. In a similar study, Loosli, Rahm et al. [4] documented more pronounced PI in older than younger adults when assessing accuracy. Subsequent fMRI data revealed that a network responsible for resolving PI, including regions such as the inferior frontal gyrus, the insula and the medial prefrontal cortex, had lower activation in older than younger adults [4].

PI in older age may also be durable and difficult to manage. A modified recent-probes task was used by Loosli, Falquez et al. [7], recruiting older adults only. PI was found on both accuracy and RT measures, but one group had daily training with the recent-probes task (and RN probes) over 10 days. Despite this, they could not entirely prevent PI, as the RT cost persisted for over a week without declining.

Heightened PI in older age is echoed in other paradigms using visual stimuli [8], and it has also been shown in verbal memory procedures, such as the AB-AC paradigm [9], the N-back task [10] and the verbal recent-probes task [11].

There are different explanations for increased susceptibility to PI in older age, but within the recent-probes task, familiarity may play a key role. According to the familiarity-inhibition model, stimuli that are familiar are more likely to prompt a “match” response, but this is problematic on RN trials, as a match response here is erroneous [2]. Attempting to inhibit the familiarity signal from RN probes can slow down responses or lead to mistakes, resulting in PI, and older adults may particularly struggle with this. According to the inhibitory-deficit hypothesis [12], the processes that allow relevant information to be maintained and irrelevant information to be ignored/deleted are weakened in older age (and in other conditions that affect attention) [13]. Reduced inhibition means older adults hold more irrelevant data in working memory, and within the context of the recent-probes task, this may mean greater susceptibility to PI due to difficulties suppressing the familiarity signal from RN probes.

An alternative explanation comes from the Dual Mechanisms of Control (DMC) framework, which hypothesizes that proactive and reactive processes contribute to cognitive control [14]. In relation to interference in working memory, proactive control would be used to anticipate and prevent interference in advance, whereas reactive control identifies and responds to interference when it happens [15]. Proactive control presents important advantages, but Xu et al. [16] have shown that older adults may be more likely to use a familiarity-driven reactive mode. This would be ineffective in the recent-probes task, as it would increase the number of errors and slow responding when familiar RN probes occur, resulting in stronger PI.

However, while the inhibitory-deficit hypothesis and DMC framework both predict increased PI in older adults, this has not always been found. For instance, Moore et al.’s [17] recent-probes task used emotionally neutral faces and scenes. On each trial, participants had to memorize three faces and three scenes over a brief delay. In different trial blocks, the probe was either consistently a face or consistently a scene, meaning one stimulus category had to be selectively memorized and the other category ignored. Responding was assessed in four ways (percent correct, *d’*, *c* and RT), and if older adults were more susceptible to PI, this would be manifested in an age group and probe type interaction. Such an interaction was not found on three of the four measures, but for RT, younger adults actually experienced higher PI than older adults.

Moore et al. [17] noted that task difficulty and cognitive load might have already slowed responding in older adults, increasing the challenge of discovering age-sensitive PI. Nonetheless, other doubts have been raised about PI in older adults. Archambeau et al. [18] applied a diffusion decision model to data from their (verbal) recent-probes task and found that the drift rate, which is linked to processing efficiency, could explain task performance. Older adults had a lower drift rate than younger adults, but there was no interaction between age and probe type, suggesting no increased vulnerability to PI in older adults. Contrary to past work, Archambeau et al. [18] concluded that the inhibitory processes used to resist PI continue to function in older age.

It is therefore uncertain if older adults really are more vulnerable to PI in VWM, but there are also important methodological issues in the recent-probes task that may influence the size of the PI effect. The recent-probes task reveals a form of item-specific PI, caused by the reoccurrence of a former target from the recent past as a current probe [19]. This represents a very selective and stimulus-specific form of PI, which differs from the broader, nonspecific PI caused by repeated exposure to a broader category of stimuli used over multiple trials [20]. However, studies using the visual recent-probes task with older adults have tended to use a small pool of stimuli that are repeated throughout the experiment—the work of Loosli and colleagues used 12 line drawings of animals, for example [3,4,7], ensuring that stimuli would be regularly repeated throughout the experiment. This might create a high level of “background” PI and introduce a form of nonspecific PI, caused by repeated exposure to stimuli over multiple trials. Indeed, in another procedure that measures PI—the Repeated-Unique Paradigm (RUP) [21]– regular exposure to a small pool of heavily repeated images impairs VWM performance in comparison to a set of unique stimuli [22,23,24].

Older adults may primarily struggle with PI when the interference context is increased. In a study using the verbal recent-probes task, Manard et al. [15] created different interference contexts by varying the proportion of RN probes, with these representing either 10% (low interference) or 40% (high interference) of trials. On the RT measure, older adults experienced greater PI in the high interference context (though this was also influenced by fluid intelligence and processing speed). Selecting stimuli from a smaller pool of items, ensuring repetition, might work in a similar manner, creating a high interference context. In a lower interference context, older adults may be better able to manage PI.

A second issue with the recent-probes task is the brief time available for responding. Typically, the probe must receive a decision within a few seconds, with prior response times including 1.5 s [4], 1.8 s [3,4] and 2 s [17]. There are two potential issues here. Firstly, RTs slow with age in both simple and complex tasks, and this may be due to difficulties processing stimuli and preparing motor movements in older age [25]. Given this, the time pressure to respond may particularly challenge older people to answer correctly. Secondly, PI may be increased when an immediate response is required. Öztekin and McElree [26] note that recognition memory involves a familiarity assessment, which happens at an early stage of retrieval, followed by the usage of more detailed episodic information at a later retrieval stage. The occurrence of RN items in the recent-probes task prevents reliable use of the fast-acting familiarity assessments, as it could lead to incorrect responses. Öztekin and McElree’s [26] results were consistent with this idea in a verbal memory paradigm, as accuracy was lower when participants had to respond quickly (as indicated by a tone) compared to when they had more time. In relation to the recent-probes task, if there is only a short period to react to the probe, participants may be forced to rely on a fast-acting but misleading familiarity assessment, heightening PI. This does seem plausible, as older adults are more likely to respond based on familiarity signals [16]. However, if more time were available to respond, additional episodic information could be used to resolve PI. In tentative support of this idea, Archambeau et al. [18] did not find substantial age-based PI in the recent-probes task when there was no time limit for responding (though this study used verbal stimuli).

Finally, a wider issue with the recent-probes task is its use of a single probe during testing. Brady et al. [27] have critiqued the use of yes/no testing (i.e., match or mismatch) as it is particularly difficult to assess accuracy while controlling response bias. While various methods that ostensibly tackle this problem do exist (e.g., *d’*, *A’*), Brady et al. [27] show that these measures have very different (potentially inaccurate) theoretical assumptions, and results that may be uncovered on one measure may be inconsistent with other measures. Instead, Brady et al. [27] recommend forced-choice testing, such as two-alternative forced-choice (2AFC), as it is theory-neutral, unbiased and more accurate. Yet in the recent-probes task, 2AFC is rarely, if ever, utilized.

Overall, the VWM of older adults may be more vulnerable to PI, but this possibility has been challenged [17,18]. Methodological issues in the recent-probes task mean it is unclear whether PI represents a specific, limited effect, or a more global, nonspecific effect due to extensive stimulus repetition. It is also plausible that PI in older adults is influenced by the need for an immediate response. Finally, the PI effect may have been skewed by a single probe recognition test, which has disadvantages [27].

The present study aimed to address these issues and reassess visual PI in younger and older adults using a modified version of the visual recent-probes task. The typical single-probe recognition test was replaced with a 2AFC task, in which a target from the present trial was paired with either an untested target from the *previous* trial (RN foil) or from three previous trials (NRN foil). PI would be represented by less accurate and/or slower responses to RN than NRN foils, and PI was estimated through a derived difference score that compared RN against NRN performance.

In one condition, the stimuli were drawn from a small pool of just eight items, ensuring regular repetition throughout the task, whereas in another condition, all targets were unique. This was inspired by the RUP [21,22,23,24]. If regular repetition heightens the wider interference context, increasing the demands of the task and making it more difficult to rely on familiarity signals, older adults should be especially vulnerable to PI, compared with younger adults and the unique condition. This would be manifested in an age group x condition interaction.

To assess whether the pre-response delay affected PI, a tone was used to indicate when a response was needed – a sound cue occurred either immediately as the 2AFC test started, or after a 3 s delay. This was inspired by Öztekin and McElree’s [26] approach. If PI in older adults is particularly affected by usage of a fast-acting familiarity signal, older adults should be more vulnerable to PI than younger adults when cued to respond immediately. However, a delay before responding might permit additional episodic information to be used, allowing older adults to better manage PI. This would be manifested in an age group × pre-response delay interaction.

## 2. Materials and Methods

### 2.1. Participants

The sample size was estimated based on the hypothesized two-way interactions in a mixed experimental design. According to Brysbaert [28], 200 participants would be needed to detect the smallest effect size of interest (*d* = 0.4) with 80% power at 0.05 significance (this sample size would also be capable of detecting main effects for factors with two levels—see Table 7 in Brysbaert [28]). Effort was made to moderately over-recruit to account for any unusable data. In total, 208 participants based in the UK or USA were recruited via Prolific Academic (https://www.prolific.com/), but one requested data to be withdrawn, and another had excessive missing data (over 30% of responses were missing).

The final sample included 102 younger adults (48 women and 54 men) aged between 18 and 29 (*M* = 22.36, *SD* = 2.16) and 104 older adults (64 women, 38 men, two undisclosed gender) aged between 64 and 80 (*M* = 69.90, *SD* = 3.83). Following Rhodes et al. [8], participants were not eligible to volunteer if they had a history of mental health issues, a diagnosis of dementia or mild cognitive impairment, or a history of head injuries. They were also required to have normal or corrected-to-normal vision. Prolific’s screening function only advertised the study to those meeting these eligibility criteria. Participants were paid £4.50 for completing the study.

### 2.2. Stimuli

Images used in the recent-probes task were sourced from Brady et al.’s [29] database, featuring photos of everyday objects and items (e.g., tools, appliances, food products, toys, etc.) and animals. They were shown on a white background, and any images relating to familiar brands or common phobias were removed. Then, 520 images were randomly selected to form the target/probe set in the unique condition. To create the repeated condition stimuli, 64 images were randomly selected and allocated into eight different sets, where each set contained eight images. The use of eight images ensured extensive target repetition throughout the task [30], and the creation of different sets meant that different groups of participants experienced different repeated stimuli, reducing concerns that artifacts of specific images would affect the results. Throughout the experimental trials, each repeated image was used 78 times as targets and probes, on average (varying between 69 and 81 times). Lastly, Audacity (version 3.7.1; https://www.audacityteam.org/) was used to create the cue that indicated when a response was needed, which was a 440 Hz pure tone lasting 500 ms.

### 2.3. Design and Procedure

The experiment was designed and built in Gorilla Experiment Builder (version 2; https://gorilla.sc) [31], with participants completing the study on a desktop computer or laptop. Each trial began with a black fixation cross presented in the center of a white background for 300 ms, followed by four to-be-remembered targets. Targets were displayed for 1.5 s in a 2 × 2 grid and were unique within a trial. After a 1 s unfilled delay, a 2AFC recognition task occurred. Here, a target presented on the current trial was paired with an untested foil item from an earlier trial. Foils were either an RN probe—an untested target from the previous trial—or an NRN probe—an untested target from Trial *N*-3. NRN probes were selected from Trial *N*-3 because (1) it has been shown that *N*-3 probes produce less PI than *N*-1 probes in the visual recent-probes task [19], and (2) it still allowed the repeated condition to feature regular occurrence of all eight stimuli. Crucially, the study was designed to ensure that no intervening exposures of stimuli contaminated the *N*-1/*N*-3 comparison—i.e., NRN and RN probes would have last been displayed as a target on Trial *N*-3 and Trial *N*-1 only, respectively. Targets and foils occupied the left and right positions in the 2AFC task an equal number of times, and each of the four target positions was equally tested too.

When the 2AFC task occurred, participants had to click the image forming part of the current target set. They were cued to respond by a tone that occurred immediately (i.e., as soon as the 2AFC task began) or after a delay of 3 s. This created two pre-response delays—no delay vs. delay—and was influenced by Öztekin and McElree’s [26] approach.

**Figure 1:**
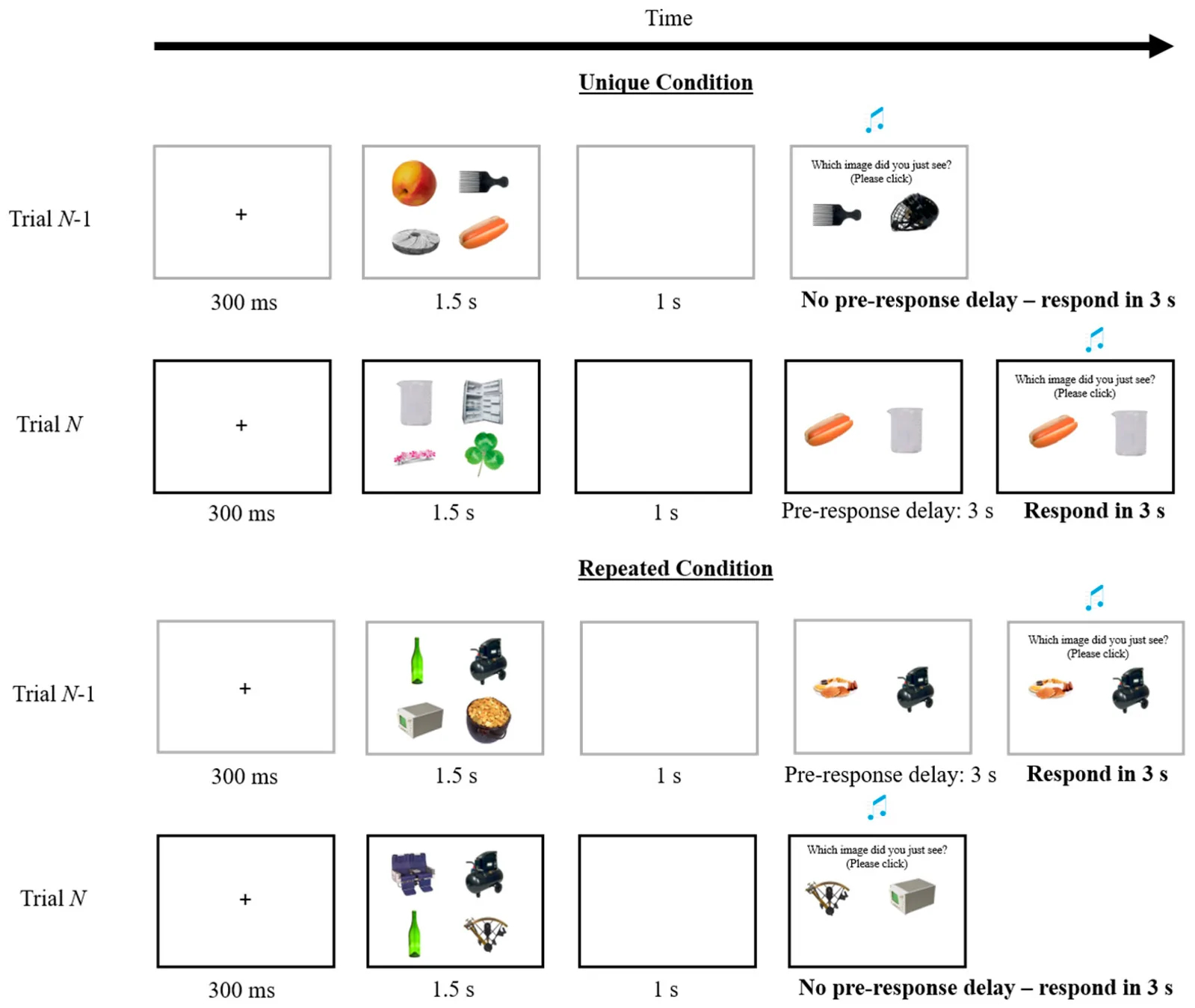
Example trials in the recent-probes task. Boxes in gray show Trial *N*-1 and boxes in black show Trial *N* (the current trial). After a fixation cross, four to-be-remembered target images were shown. After an unfilled delay, the 2AFC began, with participants needing to click on the image they believed was part of the Trial *N* target set. A tone indicated when they should respond, along with a written instruction presented at the same time as the cue. The top two rows show the unique condition. Trial *N*-1 shows an NRN foil without any pre-response delay (immediate response), whereas the second row shows an RN foil and a delayed response. The bottom two rows show the repeated condition, with stimuli from Set A. Row 3 shows an NRN foil and a delayed response, whereas row 4 shows an RN foil and an immediate response.

Once the tone sounded, participants had 3 s to respond, and a countdown timer at the bottom of the screen indicated the time available for the selection. Above the two images, the phrase “Which image did you just see? (Please click)” appeared, but this was only presented when the participant could actually respond, to make it clearer when a response was required. After participants responded or after 3 s had elapsed, the next trial began after a 1 s delay. See Figure 1 for a diagram of the procedure.

A mixed experimental design was used, with the between-groups factor being age group (young vs. old) and the within-groups factors including condition (repeated vs. unique), pre-response delay (no delay vs. delay) and foil type (RN vs. NRN). Several additional controls were put in place. True randomization of trials in the recent-probes task is not possible, as there is a critical relationship between successive trials. Instead, trials were organized into eight mini-blocks, with each block containing 13 trials (12 experimental trials and a “warm-up” trial). These had a fixed structure, but the mini-block order was randomized. The warm-up trials started each block and were designed to introduce a stimulus that would be used as a foil on the next trial.

The repeated and unique conditions were completed within separate blocks (containing all eight mini-blocks), but the order of the two conditions was counterbalanced. Participants were also randomly allocated to receive one of the eight different stimulus sets comprising the repeated condition.

In total, participants completed 104 trials for each condition, including eight warm-up trials. The 96 experimental trials were divided equally between 48 trials with no pre-response delay and 48 with a delay (24 with an RN probe and 24 with a NRN probe).

Prior to the experimental task, participants viewed a short video providing task instructions and then completed eight practice trials (four requiring an immediate response and four requiring a delayed response). Stimuli used on the practice trials came from the repeated condition, providing early exposure to those images that was designed to heighten their familiarity (and potentially PI).

At the end of the procedure, participants had an option to submit or withdraw their data, after which they received a debriefing. Full information about the experimental trials can be accessed at https://doi.org/10.17605/OSF.IO/GPCRW (accessed on 6 August 2026), and the tasks and study created in Gorilla Experiment Builder are available at https://app.gorilla.sc/openmaterials/1304088 (accessed on 25 June 2026).

## 3. Results

### 3.1. Statistical Analysis

Trials with missing responses were excluded, but these were rare (on average, 1.2% of trials did not receive a response within the 3 s period), and warm-up trials were removed. Analyses on RT were based on trials with a correct response only. Some responses could be very fast when there was a pre-response delay present, as participants were already waiting to respond, so no specific cut-off was used to remove RTs. However, in trials without a delay, responses in under 100 ms were very rare (representing 0.05% of trials).

To formally analyze PI effects, frequentist and Bayesian ANOVAs were used, with post hoc tests utilized when required. The *p*-values for any post hoc tests were corrected using the Bonferroni adjustment. Frequentist and Bayesian *t*-tests were also conducted as part of the preliminary assessment and to follow up on some interactions. Additionally, frequentist and Bayesian correlation was used to assess the relationship between task accuracy and RT. These analyses were performed in JASP (Version 0.18.1; [32]). Bayesian correlations, *t*-tests and one-way ANOVAs generated a BF_10_ score, where values of 3, 10 or 100 indicate moderate, strong, and extreme evidence for the alternative hypothesis over the null hypothesis, respectively. Values ranging between 1 and 3 are typically classed as insensitive (offering only anecdotal evidence for the alternative hypothesis), and those below 0.33 indicate more support for the null than alternative hypothesis [33].

The factorial Bayesian ANOVA used BF_Inclusion_ values and matched models to assess whether data were more likely under models with one effect than without (e.g., whether a model with an interaction had more support than a model without that interaction [34]). BF_Inclusion_ values can be interpreted in the same way as BF_10_ values, using the same thresholds as outlined above, but they assess evidence for retaining a particular effect/interaction in the model [33]. I.e., a BF_Inclusion_ value of 10 would provide strong evidence for including a specific effect/interaction in the model, whereas a value of 0.1 provides strong evidence against including a specific effect/interaction. As few studies have used Bayesian analysis to assess responses in the recent-probes task, default priors provided in JASP were employed. These default priors, based on a Cauchy distribution, are intended to be general, applicable to a wide range of scenarios and computationally convenient [35].

These analyses were supplemented with mixed models. A generalized linear mixed model (GLMM) was used to assess task accuracy, using the glmmTMB package (version 1.1.14; [36], and it was based on a binomial distribution and logit link function. A linear mixed-effect model was used to assess RT via the lme4 package (version 2.0-6; [37]. Interactions were followed up with the emmeans package (version 2.0.4), and both mixed models were conducted in RStudio (version 2026.05.0; [38]).

### 3.2. Preliminary Analyses

Firstly, to assess whether the repeated stimulus set affected performance, one-way frequentist and Bayesian between-groups ANOVAs were conducted on overall task accuracy (proportion correct) and RT for the repeated conditions. There was no effect on accuracy, *F*(7, 198) = 1.59, *p* = 0.142, η_p_^2^ = 0.05, BF_10_ = 0.19, or RT, *F*(7, 198) = 0.70, *p* = 0.671, η_p_^2^ = 0.02, BF_10_ = 0.03. Additionally, frequentist and Bayesian independent-samples *t*-tests assessed whether the condition order (repeated-unique or unique-repeated) influenced overall task accuracy and RT. In both cases, there was no effect (accuracy: *t*[204] = −0.44, *p* = 0.661, *d* = −0.06, BF_10_ = 0.17; RT: *t*[204] = 0.83, *p* = 0.406, *d* = 0.12, BF_10_ = 0.21).

Secondly, the presence of PI was assessed. Performance on trials with RN and NRN foils was collapsed across condition and pre-response delay, and contrasted with paired-samples frequentist and Bayesian *t*-tests. For accuracy, there was a significant PI effect, *t*(205) = 10.15, *p* < 0.001, *d* = 0.71, with extreme support for the alternative hypothesis (BF_10_ = 6.28 × 10^16^). This was caused by fewer correct responses when the foil matched an item from the previous trial (RN *M* = 0.86) than in the three prior trials (NRN *M* = 0.89). Likewise, there was a significant PI effect on the RT measure and extreme support for the alternative hypothesis, *t*(205) = −4.22, *p* < 0.001, *d* = −0.29, BF_10_ = 359.26, with slower responses on RN (*M* = 902.67 ms) than NRN trials (*M* = 886.35 ms).

### 3.3. PI Effects on Accuracy

As PI was present, a score to represent PI was calculated by subtracting RN from NRN trials, and descriptive statistics are shown in Table 1. Values above zero indicated PI. The PI effect, based on the NRN-RN contrast, was generally modest, except for the unique condition and no pre-response delay, where much more notable PI was observed.

The PI scores were assessed for normality and homogeneity of variance. Skewness and kurtosis scores were within the acceptable range [39], and a histogram and a Q-Q plot did not highlight major deviations from normality, but Levene’s test suggested a potential issue with homogeneity of variance in the repeated condition with a delayed response (*p* = 0.026). However, the range of standard deviations was low (older adults *SD* = 0.094; younger adults *SD* = 0.076) and the variance ratio was only 1.53, well below the 1:4 ratio suggested as problematic [40]. A residual-versus-fitted plot did not suggest issues with heteroscedasticity either, so the significant Levene’s result may have been due to the large sample size. Given this, data were assumed to meet parametric assumptions, but a robust statistical test supported the main conclusions outlined below.

Next, 2 (age group: younger vs. older adults) × 2 (condition: repeated vs. unique) × 2 (pre-response delay: no delay vs. delay) mixed frequentist and Bayesian ANOVAs were conducted on the PI accuracy score.

There was a significant main effect of condition, *F*(1, 204) = 5.05, *p* = 0.026, η_p_^2^ = 0.02, with more PI in the unique (*M* = 0.04) than repeated (*M* = 0.03) condition, though the Bayesian ANOVA showed little support for retaining this main effect in the model (BFInclusion = 0.69).

There was also a significant and strongly supported main effect of pre-response delay, *F*(1, 204) = 64.47, *p* < 0.001, η_p_^2^ = 0.24, BF_Inclusion_ = 6.03 × 10^9^, with greater PI when there was no pre-response delay (immediate response: *M* = 0.06; delayed response: *M* = 0.01). Importantly, age group did not affect the overall PI score, *F*(1, 204) = 0.82, *p* = 0.368, η_p_^2^ = 0.004, BF_Inclusion_ = 0.14 (older adults: *M* = 0.04; younger adults: *M* = 0.03).

Two of the interactions were non-significant and unsupported, including condition × age group, *F*(1, 204) = 0.24, *p* = 0.627, η_p_^2^ = 0.001, BF_Inclusion_ = 0.15, and the three-way interaction, *F*(1, 204) = 0.14, *p* = 0.709, η_p_^2^ < 0.001, BF_Inclusion_ = 0.25.

**Figure 2:**
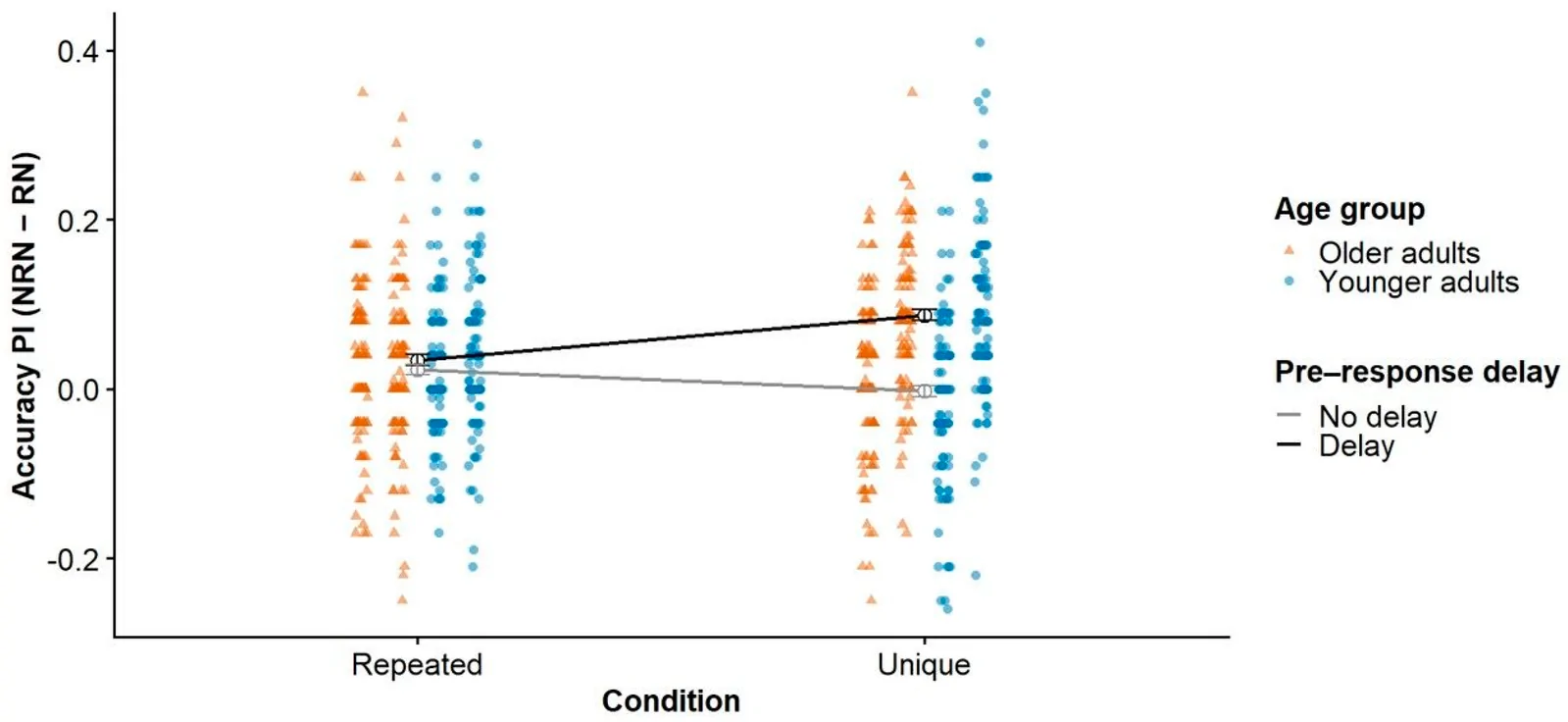
Interaction between pre-response delay and condition for the accuracy PI measure. Repeated and unique conditions are shown on the horizontal axis; trials involving an immediate response are shown by the black line and trials involving a delayed response are shown by the gray line. Circles represent the mean with standard error within. Jitter points show individual participant scores according to age.

For condition × pre-response delay, there was a significant interaction and strong evidence for retaining it within the model, *F*(1, 204) = 36.46, *p* < 0.001, η_p_^2^ = 0.15, BF_Inclusion_ = 2.92 × 10^8^. Bonferroni post hoc tests and Bayesian paired-samples *t*-tests showed no difference in PI between pre-response delays in the repeated condition, *t*(205) = −1.42, *p*_Bonferroni_ = 0.628, *d* = −0.10, BF_10_ = 0.21, but there was notably higher PI in the unique condition when the there was no pre-response delay, *t*(205) = −9.15, *p*_Bonferroni_ < 0.001, *d* = −0.64, BF_10_ = 9.04 × 10^13^. In the unique condition with a delayed response, PI was not present and so was lower than PI in the equivalent repeated condition, *t*(205) = 2.94, *p*_Bonferroni_ = 0.016, *d* = 0.21, BF_10_ = 5.06. In contrast, when there was no pre-response delay, PI in the unique condition exceeded that in the repeated condition, *t*(205) = −5.68, *p*_Bonferroni_ < 0.001, *d* = −0.40, BF_10_ = 201,483.42. See Figure 2.

For pre-response delay × age group, there was a conventionally significant interaction, *F*(1, 204) = 6.05, *p* = 0.015, η_p_^2^ = 0.03, though the Bayesian ANOVA suggested limited support for retaining the interaction in the model (BF_Inclusion_ = 1.73) and Bonferroni post hoc tests did not find significant differences between younger and older adults in each condition.

A binomial GLMM was then conducted on trial-level accuracy scores (where individual responses were classed as correct or incorrect). Participants were classed as a random intercept to account for repeated, non-independent observations within participants, and to manage baseline accuracy. Replicability of effects from the ANOVA could then be assessed. The model included the three previous factors, while incorporating foil type (RN or NRN) as a fourth factor. The GLMM has the advantage of retaining the full set of individual responses, rather than an aggregated effect, and has better statistical power [41]. Model convergence was confirmed, and further diagnostics were assessed with the DHARMa package (version 0.5.0; [42]. This showed that there was no evidence of non-uniform residuals, overdispersion or excessive outliers.

The outcomes of the GLMM are shown in Table 2. Matching the frequentist and Bayesian ANOVA above, age group was not significant and did not interact with any of the other factors. The effect of condition was significant, and in this case represented poorer overall performance in the unique than repeated condition. Pre-response delay did not significantly affect overall accuracy (though it did interact with some other factors). The newly incorporated foil type variable was significant and reflected PI, with fewer correct responses to RN than NRN trials. The pre-response delay × condition interaction was significant for overall accuracy, and foil type also interacted with condition. However, both interactions were subsumed within a higher-order interaction between foil type, pre-response delay and condition.

To assess the three-way interaction, estimated marginal means were calculated, and RN and NRN foils were compared within each condition. In the repeated condition, PI was present for both types of pre-response delays (no delay: *Z* = 5.42, *p*_Bonferroni_ < 0.001, Odds Ratio/OR = 1.44 [95% *CI*: 1.26, 1.64]; delay: *Z* = 3.79, *p*_Bonferroni_ < 0.001, OR = 1.29 [95% *CI*: 1.13, 1.48]). This was due to worse performance on RN than NRN trials. In the unique condition, PI was absent when the response was delayed (*Z* = −0.54, *p*_Bonferroni_ = 0.999, OR = 0.97 [95% *CI*: 0.86, 1.09]), but it was substantial when there was no pre-response delay (*Z* = 12.82, *p*_Bonferroni_ < 0.001, OR = 2.28 [95% *CI*: 2.01, 2.59]), as observed in Figure 2.

### 3.4. PI Effects on RT

To create a variable to represent PI for RT, NRN RTs were subtracted from RN RTs, so again values above zero indicated PI. Descriptive statistics are shown in Table 3. PI was absent in the repeated condition when the response was delayed (and also in younger adults in the unique condition with a delayed response), but it was more notable when there was no pre-response delay.

The PI scores on RT were assessed for normality and homogeneity of variance. Levene’s test was consistently non-significant, and all skewness/kurtosis scores were within acceptable ranges [39]. A histogram and Q-Q plot did not highlight major concerns about normality either, though there may have been some minor deviations from normality in the tails.

Three-way frequentist and Bayesian mixed ANOVAs found only one main effect. There was greater PI when there was no pre-response delay (no delay: *M* = 32.57 ms; delay: *M* = 0.06 ms), *F*(1, 204) = 21.85, *p* < 0.001, η_p_^2^ = 0.10, BF_Inclusion_ = 933.68.

There was a marginally significant effect of condition, *F*(1, 204) = 3.73, *p* = 0.055, η_p_^2^ = 0.02, with greater PI in the unique (*M* = 23.57 ms) than repeated (*M* = 9.07 ms) conditions, but this was unsupported and insensitive in the Bayesian analysis (BF_Inclusion_ = 0.57).

The main effect of age group was non-significant and unsupported, *F*(1, 204) = 0.09, *p* = 0.763, η_p_^2^ < 0.001, BF_Inclusion_ = 0.11, with a very similar PI effect in younger (*M* = 15.15 ms) and older (*M* = 17.49 ms) adults.

All interactions were also non-significant and unsupported: Age group × condition, *F*(1, 204) = 1.24, *p* = 0.268, η_p_^2^ = 0.01, BF_Inclusion_ = 0.19; age group × pre-response delay, *F*(1, 204) = 3.09, *p* = 0.080, η_p_^2^ = 0.02, BF_Inclusion_ = 0.53; condition × pre-response delay, *F*(1, 204) = 0.97, *p* = 0.325, η_p_^2^ = 0.01, BF_Inclusion_ = 0.19; age group × condition × pre-response delay, *F*(1, 204) = 0.01, *p* = 0.925, η_p_^2^ < 0.001, BF_Inclusion_ = 0.13.

Finally, a linear mixed-effect model was used to assess RTs while retaining individual trial-level information. A similar approach was adopted to the GLMM for accuracy, with participant added as a random intercept, to account for non-independent, repeated observations and potential baseline differences in responding not addressed by the ANOVA. The three original factors along with foil type were included. The linear mixed-effect model was used as RT scores were continuous, but they were log-transformed due to evidence of a positive skew [43]. Fixed effects were assessed using Type III ANOVA based on Satterthwaite’s approach for approximating degrees of freedom [44]. Model convergence was achieved, though there remained some departures from normality, with some mild heteroscedasticity. This may have been influenced by trials with a pre-response delay, where RTs could be very fast after the waiting period (likely because participants could prepare their response). As such, these issues were not of concern, especially given the size of the data set (over 34,000 observations).

As seen in Table 4, all main effects were significant. PI was present, as responding to RN trials was slower than NRN trials. Older adults were slower to respond than younger adults and, as expected, RTs were faster when there was a delay before responding, rather than no delay. Responses were also quicker in the unique than repeated condition.

All two-way interactions were significant, except for foil type × pre-response delay, which was marginal. However, there were some three-way interactions that subsumed the other interactions, and these were the focus of the follow-up tests.

For age group × pre-response delay × condition, the condition effect was explored separately according to age and pre-response delay. For older adults, responses in the repeated condition were slower than the unique condition for trials with a delay (*Z* = 3.60, *p*_Bonferroni_ = 0.001) and without one (*Z* = 6.11, *p*_Bonferroni_ < 0.001). Younger adults were also slower to respond in the repeated than unique condition when there was no pre-response delay (*Z* = 3.05, *p* = 0.009). However, for delayed responses, the reverse effect emerged, with faster responses in the repeated than unique condition (*Z* = −4.86, *p*_Bonferroni_ < 0.001).

For the age group × foil type × pre-response delay interaction, RN and NRN trials were compared for each age group and pre-response delay, to specifically assess PI. When there was no delay to the response, younger adults showed the standard PI effect (*Z* = −2.87, *p*_Bonferroni_ = 0.016), but not when the response was delayed (*Z* = 1.08, *p*_Bonferroni_ = 0.999). No differences were found between RN and NRN trials according to pre-response delay for older adults when *p* values were corrected (delay: *Z* = −2.11, *p* = 0.140; no delay: *Z* = −2.01, *p* = 0.176).

### 3.5. Correlating Accuracy and RT

As older adults were notably slower at responding than younger adults, but not less accurate, a speed-accuracy trade-off may have occurred in older adults. To assess this, frequentist and Bayesian Pearson correlations assessed the relationship between overall task accuracy and overall RT. In older adults, there was a significant positive correlation between accuracy and RT, *r*(102) = 0.20, *p* = 0.048, though the Bayes factor was insensitive (BF_10_ = 0.85). In younger adults, there was a marginal relationship between accuracy and RT, but this was in a negative direction, *r*(100) = −0.18, *p* = 0.073. The correlation was insensitive from a Bayesian perspective (BF_10_ = 0.60).

## 4. Discussion

The present study reassessed whether older adults are more vulnerable than younger adults to PI in VWM. A well-established method for assessing PI, the recent-probes task, was used. While some previous studies have found age-sensitive PI [3,4], there is inconsistent evidence [17,18], and potential features of the recent-probes task—especially the need for an immediate response (no pre-response delay), the use of a small pool of stimuli, and the yes/no recognition task—could plausibly exaggerate any PI effect in older adults.

PI was found on both accuracy and RT measures here, and at both an aggregated level and individual trial level. Additionally, this study showed that PI can affect VWM in a 2AFC recognition task. The effect therefore generalizes beyond the yes/no (match/mismatch) arrangement typical in the recent-probes task. The PI effect was also more pronounced when responding was rushed via an immediate cue to respond, and less impactful when there was a delay before responding. This is consistent with the view that PI arises from a rapid assessment of familiarity [26] – when the response was immediately required, a fast-acting familiarity signal may have been used, increasing PI, whereas when more time was available to use additional episodic details via a delayed response, PI declined. While plausible, the role of familiarity was not directly tested, and there are alternative explanations in need of testing. For instance, given that encoding time and retention interval were fixed in length, the rate of presentation of stimuli and events was slower when the response was delayed, which could assist performance (although the pre-response delay did vary within trial blocks and was therefore not overly predictable).

Crucially, there was little evidence that older adults experienced greater PI than younger adults. When considering the aggregated PI effect, its impact on accuracy and RT measures was very similar in both age groups, representing very small effect sizes that were consistent with the null hypothesis. The one exception was a significant interaction between pre-response delay and age group, but post hoc analyses did not reveal differences between younger and older adults, and the Bayesian analysis found limited evidence for retaining this interaction. At an individual trial level, mixed models did not find age to impact PI in terms of accuracy, despite the increased power of this analysis. For RT, an interaction did emerge between age group, foil type and pre-response delay, but follow-up analyses did not find compelling evidence that older adults experienced greater PI – indeed, the only significant PI effect within that interaction was for younger adults responding without a pre-response delay.

The present results are therefore more consistent with studies that have not found substantial age-based differences in PI [17,18], questioning whether PI plays a major role in the age-related declines seen in VWM [1]. These results may also pose problems for the inhibitory-deficit hypothesis, which would predict older age to lead to greater difficulty managing interference, though inhibitory control was not directly measured here and it is therefore not possible to directly address the role that inhibition played in this task. Nonetheless, the absence of substantial age-based PI is particularly important as some conditions were designed to enhance PI—namely regular repetition of stimuli and no pre-response delay.

PI was enhanced when the response was immediately required, requiring a rapid reaction, but primarily in the unique condition. Stimulus repetition throughout the experiment did not produce substantial PI as expected based on the RUP [21,22,23,24], and the mixed models found more errors in the unique than repeated condition, though responding was faster overall in the former condition. However, the present study did not use the standard version of the RUP, in which foils in the unique condition have never been experienced before, and there is other work suggesting that repetition of stimuli within the recent-probes task can be beneficial [30] or have little impact, at least for meaningful stimuli [45]. Another study found that higher interference contexts can actually increase performance [46].

It is still notable that older adults were able to manage PI to a similar level to younger adults in the repeated condition. Extensive stimulus repetition was predicted to be harmful for older adults, due to additional familiarity from RN probes. As this did not happen, frequent exposure to repeated stimuli may have created robust long-term memories that aided VWM performance. In contrast, the single usage of unique targets may have reduced any involvement of long-term memory. Regular repetition may have also allowed the use of other strategies (e.g., verbal labeling) to assist in the task.

Critically, the ability of older adults to use repetition in a facilitatory way might suggest engagement with proactive control. While older adults may be expected to rely more on reactive control, there is evidence that older adults might be able to use proactive control in some circumstances. Xu et al. [16] used a double retro-cue paradigm and showed that older adults could control PI when stimuli were regularly repeated through the experiment, eventually equaling the performance of younger adults. They interpreted this through the DMC framework, where frequent stimulus repetition can eventually permit proactive control. An equivalent effect may have happened in the repeated condition here. In contrast, proactive control may have been harder to employ in the unique condition, especially when the response was rushed, which may have led to a familiarity-based reactive mode being employed. Yet this applied to both younger and older adults, suggesting they experienced similar difficulties. However, it is important to note that proactive control was not directly measured in this experiment, and this interpretation remains speculative.

The overall pattern of results in this experiment did not uncover robust age-based differences in PI, though wider assessment of task performance via the mixed models (which did not solely assess PI) did find some age-based differences in RT, with older adults being much slower to respond than younger adults, but not less accurate. Correlations revealed a positive relationship between accuracy and RT for older adults, and a negative relationship in younger adults. This is consistent with the notion that older adults are more likely to be accurate when taking longer to respond, yet the correlations were insensitive from a Bayesian perspective, and the effect for younger adults was non-significant. Further work into the strategies that younger and older adults use to manage PI would be worthwhile.

While the present study did not find age-sensitive PI, other studies have, including in the visual recent-probes task [3,4]. A key difference between the present study and these prior experiments is the recognition task – the current study used a 2AFC task whereas the work of Loosli and colleagues used a single probe [3,4], which is more typical in the recent-probes task. Yet Moore et al. [17] also used a single probe and did not find age-dependent PI, and the present study revealed PI on both accuracy and RT measures. As such, the retrieval test itself cannot fully explain the absence of age-sensitive PI.

A closer examination of the most similar previous studies indicates that the critical interaction between probe type and age group, which would demonstrate age-based differences in PI, is not overly robust. The interaction was significant on the accuracy measure for Loosli et al.’s [3] study (which also included children), but it was only marginally significant on the RT measure. In Loosli, Rahm et al. [4], the interaction was marginal for accuracy and non-significant for RT, and it was consistently non-significant for Moore et al. [17], except for RT, where younger adults experienced a greater PI cost than older adults.

Even so, the broader literature generally suggests that older adults are more vulnerable to PI [9,10,11]. These studies have tended to use verbal stimuli and other paradigms; however, they may be tapping into different forms of PI. For instance, the N-back task has been used to find age-sensitive PI [10], but participants must actively update their working memory, holding and then discarding individually presented items. This differs from the recent-probes task, where events on a previous trial are no longer relevant. PI may behave differently according to the maintenance requirements of the task (see also [47,48] for a conception of active/passive processes and links to PI).

Future work could address these possibilities by incorporating a range of different paradigms and stimuli into a single study. This would allow mechanisms underpinning different forms of PI to be directly compared. While Loosli et al. [3] used both N-back and the recent-probes tasks, there is scope to take this further, utilizing multiple PI procedures within one VWM experiment.

Further exploration of the effect of the pre-response delay would also be beneficial. As noted above, the current study had a fixed encoding time and fixed retention interval for all trials, so it was not possible to fully assess the impact of the rate of stimulus presentation and its impact on the pre-response delay. This could be more fully tested in a subsequent experiment, varying both encoding time and retention interval, alongside pre-response delay, and measuring the impact on PI.

Future work may also be able to address some of the limitations in the current study, including the use of online testing. This reduces environmental control compared to laboratory-based experiments, but overall performance was very good (87.4% of responses were correct, on average), and online testing allowed a much larger sample to be tested than is typically the case in the recent-probes task. Yet the older adults recruited here may have been more cognitively capable than some of their peers. All participants were recruited via Prolific, and the older adults engaged with this platform may be more technologically proficient than older adults not registered with this service. A meta-analysis suggests that usage of technology in older age is linked to reduced risk of problems in cognition [45], so future work could assess whether technological engagement links to PI. Ultimately though, treating older adults as a single, homogeneous group may itself be problematic, and there may only be greater sensitivity to PI within some older adults.

A second issue was that independent measures of cognitive ability were not obtained (selection was achieved via the in-built screening within the Prolific platform). This was due to time constraints of the study, and the desire to avoid introducing issues caused by task fatigue. However, extra cognitive testing, including a baseline measure of VWM, plus independent assessments of vision (including color vision deficiencies) and recording extra demographic factors, would have been advantageous. Yet the mixed model did show that accuracy on the task was similar for younger and older adults, and this may indicate that VWM ability itself may link to PI – Loosli and colleagues found age-sensitive PI in a sample where older adults had poorer overall performance than younger adults [3,4]. The degree to which overall VWM ability affects PI in older age therefore needs further testing.

Finally, the practice trials in this study introduced participants to the repeated stimulus set that would later be used on experimental trials. This was designed to further heighten familiarity with the repeated set, but it may also have provided additional learning that aided performance in the repeated condition, in contrast to the usually detrimental effect of repetition found in the RUP [21,22,23,24]. While the practice trials only formed a small part of the total trial numbers (4% of the total), it is a potential limitation of the study. More thoroughly exploring how PI changes within conditions, especially the repeated condition, over the experiment would be highly informative, though this would require a greater number of trials than used in the current experiment.

## 5. Conclusions

In conclusion, PI was found to affect VWM, especially when unique stimuli were used, and an immediate response was required. Yet there was less evidence that older adults were more vulnerable to PI than younger adults, at least the item-specific form of PI assessed in the recent-probes task in this sample.

## Figures and Tables

**Table 1:** Mean (SD) NRN proportion correct, RN proportion correct and PI cost according to condition and pre-response delay.

Age Group	Repeated/Delayed			Repeated/No Delay			Unique/Delayed			Unique/No Delay		
	NRN	RN	PI	NRN	RN	PI	NRN	RN	PI	NRN	RN	PI
Older adults	0.91 (0.10)	0.88 (0.11)	0.03 (0.09)	0.91 (0.11)	0.88 (0.10)	0.03 (0.10)	0.86 (0.10)	0.85 (0.09)	0.01 (0.10)	0.91 (0.08)	0.83 (0.09)	0.08 (0.09)
Younger adults	0.90 (0.13)	0.88 (0.13)	0.01 (0.08)	0.90 (0.13)	0.86 (0.13)	0.04 (0.09)	0.84 (0.14)	0.86 (0.12)	-0.02 (0.10)	0.91 (0.11)	0.82 (0.13)	0.09 (0.10)

**Table 2:** Summary of fixed effects from the GLM assessing trial-level accuracy.

Effect	Log-odds Estimate	*SE*	*Z*
Age group	−0.04	0.14	−0.25
Foil type	−0.34	0.10	−3.59 ***
Pre-response delay	−0.01	0.10	−0.10
Condition	−0.57	0.09	−6.11 ***
Age group × Foil type	0.17	0.14	1.28
Age group × Pre-response delay	−0.005	0.14	−0.03
Foil type × Pre-response delay	0.02	0.14	0.17
Age group × Condition	0.002	0.13	0.02
Foil type × Condition	0.26	0.13	2.06 *
Pre-response delay × Condition	0.53	0.14	3.89 ***
Age group × Foil type × Pre-response delay	−0.26	0.19	−1.36
Age group × Foil type × Condition	0.06	0.18	0.32
Age group × Pre-response delay × Condition	0.18	0.19	0.92
Foil type × Pre-response delay × Condition	−0.71	0.18	−3.88 ***
Age group × Foil type × Pre-response delay × Condition	−0.09	0.26	0.33

Note: * *p* < 0.05; ***p < 0.001.

**Table 3:** Mean (SD) RT in ms for NRN, RN and PI cost according to condition and pre-response delay.

Age Group	Repeated/Delayed			Repeated/No Delay			Unique/Delayed			Unique/No Delay		
	NRN	RN	PI	NRN	RN	PI	NRN	RN	PI	NRN	RN	PI
Older adults	551.25 (220.79)	550.91 (227.21)	−0.34 (88.14)	1448.21 (275.57)	1460.64 (274.04)	12.44 (119.88)	512.30 (156.80)	527.31 (164.16)	15.01 (76.40)	1352.12 (252.95)	1394.91 (246.27)	42.79 (118.75)
Younger adults	469.50 (222.23)	462.28 (205.51)	−7.22 (97.06)	1154.47 (277.04)	1185.80 (285.11)	31.33 (124.91)	485.87 (226.53)	478.21 (220.26)	−7.26 (92.25)	1111.24 (264.20)	1154.92 (272.59)	43.69 (110.35)

**Table 4:** Summary of fixed effects from the linear mixed-effect model assessing log-transformed RTs.

Effect	*F*	*p*
Age group	32.57	<0.001 ***
Foil type	8.71	0.003 **
Pre-response delay	46,170.16	<0.001 ***
Condition	15.37	<0.001 ***
Age group × Foil type	1.30	0.254
Age group × Pre-response delay	17.99	<0.001 ***
Foil type × Pre-response delay	3.79	0.052
Age group × Condition	32.78	<0.001 ***
Foil type × Condition	6.42	0.011 *
Pre-response delay × Condition	27.43	<0.001 ***
Age group × Foil type × Pre-response delay	4.14	0.042 *
Age group × Foil type × Condition	0.59	0.441
Age group × Pre-response delay × Condition	7.48	0.006 **
Foil type × Pre-response delay × Condition	0.18	0.669
Age group × Foil type × Pre-response delay × Condition	0.72	0.395

Note. The analysis shows results from a Type III ANOVA based on Satterthwaite’s approach for approximating degrees of freedom. * *p* < 0.05; ** *p* < 0.01; ***p < 0.001.

## Data Availability

The original data presented in the study are openly available in the Open Science Framework project “Age and Proactive Interference in Visual Working Memory: Reassessing the Recent-Probes Task” at https://doi.org/10.17605/OSF.IO/GPCRW (accessed on 6 August 2026).

## Abbreviations

The following abbreviations are used in this manuscript:

2AFC	Two-alternative forced-choice recognition test
BF	Bayes factor
*CI*	Confidence interval
DMC	Dual Mechanisms of Control framework
GLMM	Generalized Linear Mixed Model
NRN	Non-Recent Negative foil
OR	Odds ratio
PI	Proactive interference
RN	Recent Negative foil
RT	Response time
VWM	Visual working memory
